# Dizziness at a Canadian tertiary care hospital: a cost-of-illness study

**DOI:** 10.1186/s40463-019-0328-9

**Published:** 2019-01-16

**Authors:** Andre Le, Daniel A. Lelli, Sasha Van Katwyk, Debora Hogan, Kednapa Thavorn, Darren Tse

**Affiliations:** 1 Department of Otolaryngology - Head & Neck Surgery, University of Ottawa, Ottawa, Canada; 2 Department of Medicine, Division of Neurology, University of Ottawa, Ottawa, Canada; 3 Clinical Epidemiology Program, Ottawa Hospital Research Institute, The Ottawa Hospital, Ottawa, Canada; 4 School of Epidemiology and Public Health, University of Ottawa, Ottawa, Canada; 5 Institute for Clinical and Evaluative Sciences (ICES UOttawa), Ottawa, Canada

**Keywords:** Dizziness, Hospital cost, Cost of illness study

## Abstract

**Background:**

In the Canadian health care system, determining overall costs associated with a particular diagnostic subgroup of patients, in this case dizzy patients, is the first step in the process of determining where costs could be saved without compromising patient care. This study is the first Canadian study that evaluates these costs at a tertiary care hospital and will allow for the extrapolation of cost data for other similar academic health science centers, regional health initiatives, and provincial healthcare planning structures.

**Methods:**

We conducted a retrospective cohort study of patients of any age presenting to The Ottawa Hospital (TOH), a tertiary care hospital, between January 1st, 2009 and December 31st^,^ 2014 with a main diagnosis of dizziness or dizziness-related disease.

De-identified patient information was acquired through TOH Data Warehouse and included a patient’s sex, age, arrival and departure dates, Elixhauser co-morbidity score, location of presentation (emergency department or admitted inpatient) presenting complaint, final diagnosis code, any procedure codes linked to their care, and the direct and indirect hospital costs linked with any admission.

We derived the mean hospital costs and 95% confidence interval for each diagnosis. We obtained the number of patients who were diagnosed with dizziness within Ontario in year 2015–16 from Canadian Institute for Health Information (CIHI). A simple frequency multiplication was performed to estimate the total cost burden for Ontario based on the cost estimate for the same year obtained from TOH. Cost data were presented in 2017 Canadian dollars.

**Results:**

The average total hospital cost per patient with dizziness for the entire cohort is $450 (SD = $1334), with ED only patients costing $359 (SD = $214). The total estimated hospital cost burden of dizziness in Ontario is $31,202,000 (95% CI $29,559,000 – 32,844,000).

**Conclusions:**

The estimated annual costs of emergency department ambulatory and inpatient dizziness in Ontario was calculated to be approximately 31 million dollars per year. This is the first step in identifying potential areas for cost savings to aid local and provincial policy-makers in allocation of health care spending.

## Background

Dizziness is a common complaint seen in the emergency department (ED), accounting for 4.4% of all ED chief complaints in the United States in 2008 [1]. In 2011, there were an estimated 3.9 million visits to U.S emergency departments for complaints of dizziness or vertigo [2].

Despite a thorough history and physical exam of the dizzy patient, the diagnosis often remains equivocal. It is known that dizziness may be caused by dysfunction of numerous different systems in the body. Therefore, diagnostic tests are frequently ordered in the evaluation of dizziness, including blood work and neuroimaging such as CT and MRI head with the goal of ruling out more serious etiologies such as stroke. These tests have been shown, however, to have low yield and are also quite costly [3–6]. A recent study showed that CT scans and MRIs accounted for nearly 500 million dollars for evaluation of dizziness in the U.S in 2011 [2]. The same study extrapolated that the U.S national costs for patients presenting with dizziness to the ED were estimated to be approximately 4 billion dollars.

Currently, there are no Canadian studies evaluating the overall emergency department ambulatory and inpatient costs of dizziness-related visits. In a resource-limited healthcare environment, determining the cost burden of dizziness is an important first step towards improved and more efficient use of healthcare resources as well as excellent quality of care. Our study aimed to do this by calculating costs at a tertiary care hospital and further extrapolating costs at a provincial level to allow cost-effective strategies to be implemented.

## Methods

We conducted a retrospective cohort study of patients of any age presenting to The Ottawa Hospital (TOH) Civic and General Campuses between January 1st 2009 and December 31st 2014 with a most responsible diagnosis of dizziness or dizziness-related disease. The diagnoses identified were: Meniere’s disease, benign paroxysmal vertigo, other peripheral vertigo, dizziness and giddiness and vertigo of central origin. These diagnoses are based on the International Statistical Classification of Diseases and Related Health Problems, 10th Revision, Canada (ICD-10-CA). Cases for which dizziness was not the main diagnosis were excluded from the study.

De-identified patient information was acquired through TOH Data Warehouse and included a patient’s sex, age, arrival and departure dates and times, Elixhauser co-morbidity score, location of presentation, presenting complaint, final diagnostic code (which had to be one of the five dizziness diagnoses), any procedure codes linked to their care, and the direct and indirect hospital costs linked with any admission. Location of presentation was divided into three groups emergency department only, admitted from emergency department or inpatient (patients who presented directly for admission without presentation to ED). Direct costs are costs that are attributed to providing direct patient care; covering all the expenses in direct functional centres including salaries, supplies and equipment amortization. Importantly, diagnostic imaging costs are included in direct costs. Indirect costs are an overhead allocation based on a percentage of the activity in the functional centre. They consist of general institutional maintenance costs not directly related to the patient as it pertains to Diagnostic Imaging/Laboratory administration, Facilities Management, Human Resources, Finance, Health Records, etc. At TOH, case costing is used to cost individual services that the patient receives. The TOH health information system can report patient-specific consumption for several individual services, such as laboratories, in-patient nursing and pharmacy. For each of these services, the hospital tracks output measures in terms of the number of workload units attributed to each patient and then estimates the cost per patient for each service by multiplying the unit cost assigned to the service by workload units consumed by patients. Of note, physician fees were not included in data available through TOH Data Warehouse. All costs were adjusted for inflation to 2017 Canadian dollars.

This retrospective cohort study presents basic patient characteristics and summary cost information according to type of diagnosis. Given that cost data are highly skewed, comparison between variables was performed using Mann-Whitney test and the significant difference was established at *p* ≤ 0.05. To facilitate future economic evaluation studies of health care interventions for dizziness or dizziness-related disease, cost data are presented as mean (standard deviation), median (inter-quartile range (IQR)), and 95% confidence interval (CI) of the sample.

The mean costs and 95% CI for each diagnosis were used to extrapolate the estimated cost of dizziness across Ontario. We obtained the number of patients who were diagnosed with dizziness within each province and territory, in the year 2015–16 from Canadian Institute for Health Information (CIHI) Discharge Abstract Database (DAD) and National Ambulatory Care Reporting System (NACRS) [7]. A simple frequency multiplication was performed to estimate the total cost burden for Ontario based on the cost estimate for the same year obtained from TOH. The CIHI data was sparse for all provinces other than Ontario, so cost burden could not reasonably be extrapolated to these areas.

This study was approved by The Ottawa Health Science Network Research Ethics Board (REB 20160709-01H).

## Results

We identified 12,186 cases that had a main diagnosis of dizziness or dizziness-related disease between January 12,009 and December 31,2014 (Table 1). The most common diagnosis was dizziness and giddiness with 8239 (67.6%) cases, followed by benign paroxysmal vertigo with 1936 (15.9%) cases, other paroxysmal vertigo with 1736 (14.3%) cases, Meniere’s disease with 230 (1.9%) cases, and vertigo of central origin with 45 (0.4%) cases. The mean age of patients was 55.9 years (SD = 19.3) and did not significantly differ by type of diagnosis. 59.3% of all patients were female. The average Elixhauser co-morbidity score for patients was 0.08 (SD = .74) and was slightly higher, 0.11 (*p* < 0.05) among patients with dizziness and giddiness indicating that this subset of patients had more comorbidities in comparison to other groups. Patients with vertigo of central origin also had a high average Elixhauser co-morbidity score, however this group of patients was small for comparison (*n* = 45).

**Table 1:** Baseline Characteristics, by dizziness diagnosis

	All Diagnoses	Meniere’s disease	Benign paroxysmal vertigo	Other peripheral vertigo	Vertigo of central origin	Dizziness and giddiness
N	12,186	230	1936	1736	45	8239
%	100%	1.89%	15.89%	14.25%	0.37%	67.61%
Age (yrs)	55.9	56.3	56.9	56.4	59.8	55.5
Sex (female, %)	59.3	60.4	62.9	59.3	71.1	58.3
Elixhauser Score	0.08	0.03	0.03	0.02	0.38	0.11

Table 2 presents the total costs to the hospital stratified by diagnosis and type of admission. The vast majority of patients presented at the ED and were not admitted to the hospital. Consequentially, the average total cost per patient for the entire cohort is $450 (SD = $1334), with ED only patients costing $359 (SD = $214). Admitted from ED patients and patients admitted directly to the hospital (inpatient only) represent 1.8% of all dizziness diagnoses, but cost significantly more due to the costs associated with inpatients (i.e. index admission, overnight stay, prolonged care). Patients admitted directly to hospital included those directly admitted to a service without any presentation to ED.

**Table 2:** Estimated Hospital Cost per Patient, by dizziness diagnosis

		All Diagnoses	Meniere’s disease	Benign paroxysmal vertigo	Other peripheral vertigo	Vertigo of central origin	Dizziness and giddiness
Total Hospital Cost	N	12,186	230	1936	1736	45	8239
	Direct Cost	$ 310.97	$ 300.15	$ 263.77	$ 300.32	$ 509.38	$ 323.52
	Indirect Cost	$ 139.04	$ 136.97	$ 123.04	$ 134.10	$ 217.91	$ 143.47
	Mean Total Cost	$ 450.01	$ 437.12	$ 386.81	$ 434.42	$ 727.29	$ 466.99
	SD	$ 1333.68	$ 712.49	$ 655.19	$ 964.34	$ 932.92	$ 1521.21
	Median	$ 317.11	$ 285.04	$281.52	$ 315.04	$ 473.09	$ 326.29
	Q1	$ 228.70	$ 205.03	$ 212.75	$ 228.19	$ 297.88	$ 235.30
	Q3	$ 443.65	$ 406.67	$ 399.51	$ 429.70	$ 698.47	$ 454.88
	*p*-value*		0.8824	0.0230	0.5991	0.1623	0.0423
Admitted from ED	N	129	2	19	23	2	80
	Mean	$ 4101.38	$ 1735.86	$ 3805.13	$ 3686.29	$ 2969.02	$ 4260.22
	SD	$ 6532.64	$ 642.22	$ 3665.74	$ 2324.23	$ 3961.14	$ 7214.41
	Median	$ 2861.56	$ 1590.21	$ 2293.12	$ 4264.65	$ 2969.02	$ 2935.57
	Q1	$ 883.11	$ 1178.98	$ 1099.23	$ 1860.48	$ 168.07	$ 853.96
	Q3	$ 5018.84	$ 2438.40	$ 5448.42	$ 4931.35	$ 5769.97	$ 5041.15
	*p*-value*		0.5283	0.8437	0.8196	0.8060	0.5668
ED Only	N	11,964	225	1908	1700	43	8091
	Mean	$ 358.77	$ 342.47	$ 328.02	$ 357.07	$ 480.63	$ 366.23
	SD	$ 213.87	$ 225.84	$ 181.41	$ 201.34	$ 288.84	$ 221.79
	Median	$ 314.45	$ 282.87	$ 277.94	$ 310.53	$ 416.52	$ 323.41
	Q1	$226.53	$ 205.03	$ 212.13	$ 226.72	$ 280.14	$ 233.65
	Q3	$ 432.80	$ 396.39	$ 391.50	$ 419.85	$ 643.61	$ 445.80
	*p*-value*		0.2483	0.0000	0.7234	0.0003	0.0000
Inpatient Only	N	93	3	9	13	0	68
	Mean	$ 6978.94	$ 4017.94	$ 3972.52	$ 5544.31	NA	$ 7644.99
	SD	$ 9991.16	$ 302.60	$ 1807.99	$ 7720.98	NA	$ 11,115.67
	Median	$ 4079.30	$ 4116.57	$ 4334.98	$ 2245.52	NA	$ 4126.79
	Q1	$ 1797.33	$ 3678.33	$ 2935.32	$ 2010.41	NA	$ 1664.68
	Q3	$ 6094.54	$ 4258.92	$ 5090.27	$ 4723.13	NA	$ 6786.39
	*p*-value*		0.6168	0.3613	0.6063	NA	0.2246

*P-value relates to the variance in the mean diagnosis’ cost compared to that of all other diagnoses

The absolute number of diagnoses has steadily increased between the 2009 and 2014, with 1647 patients diagnosed in the first year, compared to 2342 patients diagnosed in the final year (Table 3). This rising trend of dizziness diagnosis appears to be uniform across all five types of diagnosis except for vertigo of central origin, however there are too few patients in that category to conclude a difference in diagnosis trend.

**Table 3:** Dizziness Diagnoses, by type, 2009–2014

Year	All Diagnoses	Meniere’s disease	Benign paroxysmal vertigo	Other peripheral vertigo	Vertigo of central origin	Dizziness and giddiness
2009	1647	31	298	230	11	1077
2010	1890	62	303	269	12	1244
2011	1959	45	310	298	5	1301
2012	2137	31	319	308	3	1476
2013	2211	35	319	320	9	1528
2014	2342	26	387	311	5	1239

The total estimated cost burden of dizziness in Ontario is $31,202,000 (95% CI $29,559,000 – 32,844,000)., of which $24,013,000 (95% CI $22,323,000 - $25,703,000) is attributed to a dizziness and giddiness diagnosis.

## Discussion

Dizziness poses a significant financial burden to the Canadian health care system. Healthcare costs are on the rise in Canada, more than doubling over the last 15 years to 242 billion dollars annually in 2017 [7, 8]. Given this current trend, it is important to identify specific disease-related costs and cost-efficient approaches to healthcare delivery.

This is the first study to determine costs associated dizziness in the ED and inpatient units at a Canadian tertiary care center, as well as extrapolating these costs on a provincial basis. In reviewing the literature, it appears that there is a paucity of disease-specific costs, including dizziness, in relation to overall Canadian health care expenditure.

The average hospital cost of dizziness per patient encounter is $450 (SD = $1333.68), the vast majority of these cases (98%) being ED only visits, costing $358 per encounter (SD = $213.87). Cases that require hospital admission, however, cost approximately $7000. Although these costs are less than those associated with conditions such as stroke, atrial fibrillation, heart failure, and fractures, they still represent a significant amount of healthcare utilization and total cost burden [9, 10].

Our cost estimates were lower than those reported in the published literature. Tehrani et al. reported that the average cost per ED dizziness-related visit in the United States to be 1004 U.S dollars with the costs increasing substantially compared to a prior national estimate performed 19 years previously [2]. The difference is likely related to differences in health care delivery, health financing systems and imaging practices, as neuroimaging accounted for 12% of their overall costs.

Our study shows that the estimated cost burden of ED and inpatient dizziness in Ontario is $31,202,000. It is important to note that this likely is an underestimate as our costs are based on hospital-level costs only as well as the fact that the cases included were only those with dizziness as the most responsible diagnosis. The cost of caring for dizzy patients in the community and outpatient basis are not easily measured, and most likely surpass the ED and inpatient estimate by many-fold. Physician fees are also not included in the data, which would account for further underestimation of total cost. However ultimately the burden to the health care infrastructure is best reflected in the hospital-level costing. Our center represents one of the largest tertiary care centers in the country with a catchment area of over 1.3 million people, over 1200 hospital beds and approximately 175,000 emergency visits per year.

There is significant variability in reported number of dizziness diagnoses between provinces most likely indicating that the quality of data collection is non-uniform across provinces (Table 4). A more uniform, comprehensive reporting system is not currently available. There was an increased number of diagnoses reported at The Ottawa Hospital over a five-year period, even after adjusting for population growth over the same period. There is not a clear clinical rationale for why dizziness incidence would be increasing in the population over a relatively short period of time, suggesting that dizziness has been historically under-reported. Since there has yet to be an observed plateau in cases, it is possible that our cost calculation is an underestimate of the number of true dizziness cases.

**Table 4:** Canada-Wide Dizziness Diagnoses, by type, 2015–16

2015–16	All Diagnoses	Meniere’s disease	Benign paroxysmal vertigo	Other peripheral vertigo	Vertigo of central origin	Dizziness and giddiness
Canada-wide	94,429	1102	16,230	6756	180	70,162
Ontario	69,336	763	12,104	4929	120	51,420
British Columbia	1304	25	239	119	3	918
Alberta	18,350	168	2937	1476	45	13,724
Saskatchewan	2796	78	482	131	5	2100
Manitoba	255	11	54	8	0	182
New Brunswick	232	6	42	14	3	167
Nova Scotia	1354	19	244	45	3	1043
Newfoundland and Labrador	147	22	25	5	0	95
Prince Edward Island	319	5	44	9	3	259
Northwest Territories	12	0	3	3	0	7
Yukon Territory	296	3	54	16	0	225
Nunavut Territory	0	0	0	0	0	0
Unspecified Province	30	3	3	3	0	22

ICD-10 data is widely used for research purposes, providing large samples of patients over extended periods of time. However, there have been numerous studies which have shown that ICD coding errors can lead to inaccurate results. For example, Seidel et al. revealed some weaknesses in the ability of the ICD-10 criteria to classify the underlying etiology of dizziness [11]. They found that many of the ICD-10 codes were unspecific and many specific vertiginous disorders such as vestibular migraine and superior semicircular canal dehiscence were missing. As well, they found some categories to be redundant and not distinguishable such as “other peripheral vertigo” and “other disorders of vestibular function.” The poor diagnostic accuracy of these codes could potentially lead to misclassification of some patients and therefore imprecise prevalence and cost estimates. Some emerging classification refinements to the upcoming ICD-11 system may help to resolve this issue, however this is an inherent limitation of our study. At present, there does not appear to be any other way that diagnostic information for dizziness can be easily collected or collated. Given that dizziness is not limited to a specific disease, we did a comprehensive search and included the most relevant diagnostic codes.

A limitation of the Ontario-wide extrapolation of costs is that the cost per event is based on a single tertiary hospital, which is very likely to be different across other types of hospitals. We felt that the size of our center would provide a reasonable reflection of other hospitals across the province to allow for extrapolation.

Determining overall costs associated with dizziness, is the first step in the process of determining where costs could be saved without compromising patient care. Examination of the costs associated with dizziness will allow one to further determine potential areas for cost savings and may aid local, provincial and federal policy-makers in allocation of health care spending. Future research should quantify the financial impact of dizziness on the Canada’s health care system associated with dizziness.

## Conclusion

This is the first study that estimated costs of tertiary care center emergency department ambulatory and inpatient care of dizziness in Ontario, which was calculated to be approximately 31 million dollars per year. Although this represents an underestimate, it is the first step in identifying potential areas for cost savings to aid local, provincial and federal policy-makers in allocation of health care spending. This could also serve as a model for other centers to explore their own dizziness data. The next step in further analyses would involve breaking down and evaluating the specific costs of dizziness to determine where cost savings would be feasible.

## References

[1] Newman-Toker D, Cannon L, Stofferahn M, Rothman R, Hsieh Y, Zee D (2007). Imprecision in patient reports of dizziness symptom quality: a cross-sectional study conducted in an acute care setting. Mayo Clin Proc.

[2] Saber Tehrani A, Coughlan D, Hsieh Y, Mantokoudis G, Korley F, Kerber K, Frick K, Newman-Toker D (2013). Rising annual costs of dizziness presentations to U.S. emergency departments. Acad Emerg Med.

[3] Wasay M, Dubey N, Bakshi R (2005). Dizziness and yield of emergency head CT scan: is it cost effective?. Emerg Med J.

[4] Lawhn-Heath C, Buckle C, Christoforidis G, Straus C (2012). Utility of head CT in the evaluation of vertigo/dizziness in the emergency department. Emerg Radiol.

[5] Colledge N (2002). Magnetic resonance brain imaging in people with dizziness: a comparison with non-dizzy people. J Neurol Neurosurg Psychiatry.

[6] Kattah J, Talkad A, Wang D, Hsieh Y, Newman-Toker D (2009). HINTS to diagnose stroke in the acute vestibular syndrome. Stroke.

[7] Discharge Abstract Database (2017). Canadian Institute of Health Information.

[8] Gandhi R, Stiell I, Forster A, Worthington J, Ziss M, Kitts J, Malik R. Health Spending | CIHI. Evaluating physician awareness of common health care costs in the emergency department. [https://www.cihi.ca/en/health-spending]. CJEM. 2017;20:1–11. 10.1017/cem.2017.43 28659219

[9] O'Reilly D, Hopkins R, Healey J, Dorian P, Sauriol L, Tarride J, Burke N, Goeree R (2013). The burden of atrial fibrillation on the hospital sector in Canada. Can J Cardiol.

[10] Woolcott J, Khan K, Mitrovic S, Anis A, Marra C (2011). The cost of fall related presentations to the ED: a prospective, in-person, patient-tracking analysis of health resource utilization. Osteoporos Int.

[11] Seidel D, Park J, Sesterhenn A, Kostev K (2018). Diagnoses of dizziness- and Vertigo-related disorders in ENT practices in Germany. Otology & Neurotology.

